# Lateral deviation of four types of epidural catheters from the lumbar epidural space into the intervertebral foramen

**DOI:** 10.1007/s00540-016-2177-2

**Published:** 2016-04-30

**Authors:** Tetsuya Uchino, Masahiro Miura, Yoshimasa Oyama, Shigekiyo Matsumoto, Chihiro Shingu, Takaaki Kitano

**Affiliations:** Department of Anesthesiology, Faculty of Medicine, Oita University, 1-1 Idaigaoka, Hazamacho, Yufushi, Oita, 879-5503 Japan; Department of Human Anatomy, Faculty of Medicine, Oita University, 1-1 Idaigaoka, Hazamacho, Yufushi, Oita, 879-5503 Japan

**Keywords:** Epidural anesthesia, Epidural catheter, Epidurography, Deviation

## Abstract

**Background:**

During epidural anesthesia, the catheter tip occasionally deviates from the epidural space into the intervertebral foramen, resulting in inadequate anesthesia.

**Methods:**

During postoperative plain radiography, iohexol was injected via the epidural catheter to determine its position and to observe the spread of the material. After exclusion of seven patients with catheters that migrated into the subcutaneous area and 25 patients with no evidence of the contrast medium, 415 patients were evaluated. We retrospectively compared patients to determine whether the incidence of deviation into the intervertebral foramen differed between four types of epidural catheters. We also investigated the load applied to the catheter tip using a Shimadzu Autograph AG-X-500 N-111 universal testing machine.

**Results:**

Deviation of the epidural catheter into the intervertebral foramen was observed in eight and 33 patients in the Hakko and Perifix Soft tip catheter groups, respectively. The incidence of deviation was higher in the Perifix Soft tip catheter group, and lower in the FlexTip Plus and Perifix FX catheter groups. A rapid increase was observed in the force exerted on the tips of the Hakko and Perifix Soft tip catheters, while the force transmitted to the tips of the FlexTip Plus and Perifix FX catheters gradually increased and then reached a plateau at a low level.

**Conclusions:**

The incidence of deviation was significantly lower with spiral-type catheters than with other types of catheters. This might be attributable to the gradual transmission of a lower level of force to the tip in spiral-type catheters.

## Introduction

When epidural anesthesia is administered during the preoperative period, the epidural catheter is occasionally placed incorrectly and migrates into a space other than the epidural space. In other cases, although the catheter enters the epidural space, its tip deviates from the intervertebral foramen, resulting in an inadequate anesthetic effect [1–7].

In recent years, spiral-type epidural catheters have been marketed and used in the clinical setting. These catheters are not prone to luminal collapse or bending, although they are highly flexible [8]. Furthermore, spiral-type catheters are unlikely to cause paresthesia or to be accidentally inserted into a blood vessel [4, 9–16].

We retrospectively evaluated and compared 447 patients who underwent epidurography after placement of an epidural catheter in the lumbar epidural space at our institution to determine whether the incidence of deviation into the intervertebral foramen would differ between four types of epidural catheters. In addition, using a Shimadzu Autograph AG-X-500 N-111 universal testing machine, we also investigated the load required to cause deviation from the intervertebral foramen when applied to the epidural catheter tip.

## Materials and methods

The procedure for epidurography after surgery was approved by the ethics committee of our institution, and written informed consent was obtained from the patients. After entering the operating room, the patient was placed in the left lateral decubitus position. After a local anesthetic was subcutaneously injected, a Tuohy needle was inserted. After the epidural space was identified using the loss-of-resistance technique, the epidural catheter was inserted approximately 5 cm into the epidural space. The insertion site was selected from Th12 to L5 according to the surgical site. The median or paramedian approach was used. For the loss-of-resistance technique, air or physiological saline was used. All of the parameters for insertion of the epidural catheter were determined by the treating anesthesiologists. When a physician with <5 years of experience performed the procedure, an attending physician carefully supervised. When plain radiography was performed to confirm the position of gauze and drains, as well as the presence or absence of foreign bodies after surgery, 5 ml of iohexol was injected via the epidural catheter to determine the position of the epidural catheter and to observe the spread of the contrast medium on frontal images, as described previously [17]. Deviation was defined as translocation of the tip into the intervertebral foramen from the epidural space based on the visualization of contrast enhancement in the paravertebral space or psoas major muscle after insertion of the epidural catheter into the intervertebral foramen. We excluded cases in which the catheter was confirmed to have migrated into the subcutaneous area and those in which the contrast enhancement could not be evaluated. Moreover, in all cases in which the epidural catheter deviated from the epidural space, the catheter was withdrawn to an appropriate length, and the imaging study was repeated to visualize the epidural space and to confirm the placement of the catheter. Because we did not recognize a difference in the deviation from the epidural space related to the type of catheter, the type of epidural catheter was not identified. Additionally, four types of catheters were considered at our institution for the trial use of epidural catheters in 447 patients who underwent surgery in the lower limbs or abdomen under a combination of general and lumbar epidural anesthesia between April 2007 and September 2011 (Table 1). The Hakko catheter (Hakko Co., a nylon block copolymer radiopaque catheter with a diameter of 1 mm and a round tip with lateral holes for continuous epidural anesthesia) was used in 86 patients from April to November 2007; the 20-gauge Perifix Soft tip catheter™ (B. Braun, a catheter made of polyamide resin and polyurethane with a soft tip and lateral holes) was used in 264 patients from December 2008 to March 2010; the FlexTip Plus™ catheter (Arrow Japan, a spiral-type 19-gauge Arrow catheter made of polyurethane [internal coil: stainless steel] with holes at the tip for epidural anesthesia) was used in 45 patients from June 2010 to March 2011; and the 19-gauge Perifix FX™ catheter (B. Braun, a spiral-type catheter made of polyamide resin and polyurethane [internal coil: stainless steel] with a round tip with lateral holes) was used in 52 patients from March to September 2011. The same type of catheter was used in all cases of epidural catheterization performed during each period.

**Table 1:** Epidural catheters

Catheters	Size	Material	Tip shape	Terminal hole or lateral eyes	Stainless steel coil
Hakko	1 mm in diameter	Nylon	Round	Lateral	−
Perifix Soft Tip	20 G	Polyamide resin, polyurethane	Soft tip	Lateral	−
FlexTip Plus	19 G	Polyurethane	Straight	Terminal	+
Perifix FX	19 G	Polyamide resin, polyurethane	Round	Lateral	+

We found that the type of epidural catheter and the deviation from the lumbar epidural space seemed to be related, and we performed a retrospective study of patients who underwent epidurography after surgery. This study was also approved by the ethics committee of our institution.

**Fig. 1:**
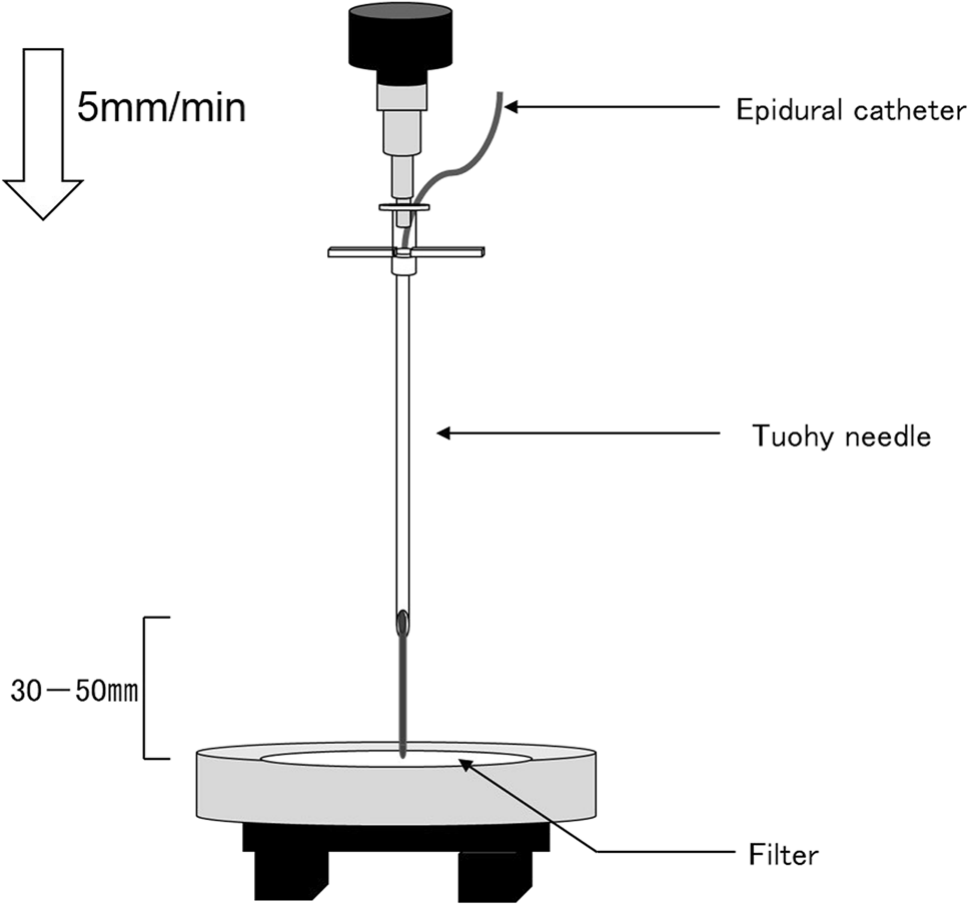
Measurement of the stress on the filter by the tip of the epidural catheter. A Shimadzu Autograph AG-X-500 N-111 universal testing machine was used to measure the force applied to a filter when an epidural catheter protruding from a Tuohy needle by 30, 40 or 50 mm was fixed to the crosshead and advanced at a speed of 5 mm/min

In our previous study using cadaver specimens, we attempted to reproduce the deviation of each type of epidural catheter into the intervertebral foramen. The Hakko and Perifix Soft tip catheters easily deviated when they were inserted into the epidural space, whereas deviation was difficult to reproduce with the FlexTip Plus and Perifix FX catheters. External force transmitted to the catheter tip has been shown to be an important factor that varies according to the catheter type [18]. Therefore, we applied a load to the tip of each of the four types of epidural catheters to determine the force required to cause deviation. The Shimadzu Autograph AGX-500 N-111 universal testing machine was used to measure the force applied to a filter with a thickness of 150 µm and a diameter of 90 mm (Merck Millipore) when an epidural catheter protruding from a Tuohy needle by 30, 40, or 50 mm was fixed to the crosshead and advanced at a speed of 5 mm/min. One catheter was used for each measurement, and five measurements were obtained and averaged for each catheter type (Fig. 1).

### Statistical analysis

The data were retrospectively compared between the four groups according to the catheter type. Regarding the patient characteristics, the Kruskal–Wallis test was performed for age and body mass index (BMI). The chi-squared independence test was performed for sex, American Society of Anesthesiologists (ASA) physical status, insertion level, loss-of-resistance results, the approach used, years of experience of the treating anesthesiologists, and incidence of deviation. Fisher’s exact test was performed to determine the probability of significance. Because the number of years of experience of the treating anesthesiologists was a covariate, for the sake of convenience, the patients were divided into those treated by anesthesiology specialists with ≥6 years of experience and those treated by residents with ≤5 years of experience (in the late stage of their postgraduate clinical training). The level of significance was determined at a *P* value of 5 %. To analyze factors affecting deviation of the epidural catheter, multiple logistic regression analysis was performed with the incidence rate of deviation as a dependent variable and each factor as an independent variable. SPSS Statistics version 20.0 for Windows (SPSS Inc., Chicago, IL, USA) was used for data analysis.

## Results

Epidurography was performed after surgery in 447 patients receiving anesthetic management with a combination of general and lumbar epidural anesthesia. After exclusion of seven patients with plain abdominal radiographs showing a catheter migrating into the subcutaneous area and 25 patients with radiographs showing no evidence of the contrast medium (10 with Hakko catheters, 11 with Perifix Soft tip catheters, two with FlexTip Plus catheters, and two with Perifix FX catheters), a total of 415 patients were compared according to the catheter type.

Between the four groups, no significant difference was observed in patient characteristics such as age, BMI, male-to-female ratio, and ASA physical status (Table 2). Although there was no significant difference in the incidence of deviation associated with the insertion site or the use of different loss-of-resistance techniques and approaches, significant differences were observed in the number of years of experience of the treating anesthesiologist (*P* < 0.05) (Table 3). Deviation of the epidural catheter into the intervertebral foramen was observed in eight patients in the Hakko catheter group and 33 patients in the Perifix Soft tip catheter group, and significant differences in the incidence rates were observed between the four groups. When the residual differences after adjustment were assessed, the incidence of deviation was significantly higher in the Perifix Soft tip catheter group and significantly lower in the FlexTip Plus catheter and Perifix FX catheter groups (Table 4).

**Table 2:** Demographic characteristics of patients

Catheters		Hakko (*n* = 74)	Perifix Soft Tip (*n* = 248)	FlexTip Plus (*n* = 43)	Perifix FX (*n* = 50)	*P* value
Sex (M:F)		30:44	73:175	13:30	15:35	0.35
Age (years)		55.9 ± 15.5	56.1 ± 16.5	50.9 ± 15.7	53.1 ± 15.6	0.21
Body mass index (kg/m^2^)		22.7 ± 3.5	23.2 ± 3.9	22.8 ± 4.0	23.0 ± 4.0	0.67
ASA physical status	1	7 (9.5 %)	51 (20.6 %)	12 (27.9 %)	12 (24.0 %)	0.12
	2	65 (87.8 %)	192 (77.4 %)	30 (69.8 %)	38 (76.0 %)	
	3	2 (2.7 %)	5 (2.0 %)	1 (2.3 %)	0 (0.0 %)	

Data are expressed as mean (±standard deviation) for continuous data and actual numbers (percentages in parentheses) for categorical data

**Table 3:** Insertion sites of the epidural catheter, epidural technique and years of experience

Catheters		Hakko (*n* = 74)	Perifix Soft Tip (*n* = 248)	FlexTip Plus (*n* = 43)	Perifix FX (*n* = 50)	*P* value
Level	Th12/L1	12 (24.0 %)	72 (36.0 %)	13 (33.3 %)	24 (49.0 %)	0.191
	L1/2	24 (48.0 %)	91 (45.5 %)	21 (53.8 %)	18 (36.7 %)	
	L2/3	11 (22.0 %)	34 (17.0 %)	4 (10.3 %)	7 (14.3 %)	
	L3/4	3 (6.0 %)	2 (1.0 %)	1 (2.6 %)	0 (0.0 %)	
	L4/5	0 (0.0 %)	1 (0.5 %)	0 (0.0 %)	0 (0.0 %)	
Loss-of-resistance technique	Saline	19 (25.7 %)	41 (16.5 %)	10 (23.3 %)	11 (22.0 %)	0.256
	Air	55 (74.3 %)	207 (83.5 %)	33 (76.7 %)	39 (78.0 %)	
Approach	Median	43 (58.1 %)	122 (49.2 %)	22 (51.2 %)	25 (50.0 %)	0.608
	Paramedian	31 (41.9 %)	126 (50.8 %)	21 (48.8 %)	25 (50.0 %)	
Experience (≤5 years: >6 years)		60:14	186:62	31:12	22:28	*P* < 0.05

Data are expressed as actual numbers (percentages in parentheses) and *P* < 0.05 for difference between catheter type

**Table 4:** The incidence of deviation into intervertebral foramen with various epidural catheters

Catheter	Epidural space	Deviation from intervertebral foramen	*P*
Hakko	66 (89.2 %)	8 (10.8 %)	<0.05
Perifix soft tip	215 (86.7 %)	33 (13.3 %)	
FlexTip plus	43 (100.0 %)	0 (0.0 %)	
Perifix FX	50 (100.0 %)	0 (0.0 %)	

Moreover, to identify factors affecting deviation, multiple logistic regression analysis was attempted with deviation as a dependent variable and sex, age, BMI, ASA physical status, insertion site, loss-of-resistance technique, approach, epidural catheter, and the number of years of experience of the treating anesthesiologist as independent variables. However, because there was no incident of deviation in the FlexTip Plus catheter group or the Perifix FX catheter group, the analysis could not be performed. Thus, a multivariate analysis was performed to compare the Perifix Soft tip catheter group, which showed a significantly higher incidence of deviation compared to the pooled results for the other categories. As a result, only the epidural catheter type yielded a *P* value of <0.05 and was found to be a significant factor for the dependent variables, with an odds ratio of 3.703.

Some data related to intravascular migration and paresthesia during catheterization could not be obtained. Intravascular migration was observed in 14 of 139 patients with the Perifix Soft tip catheter, in one of 41 patients with the FlexTip Plus catheter, and in one of 49 patients with the Perifix FX catheter (*P* = 0.088). Paresthesia during catheterization was observed in 16 of 138 patients with the Perifix Soft tip catheter, in five of 41 patients with the FlexTip Plus catheter, and in two of 49 patients with the Perifix FX catheter (*P* = 0.315).

**Fig. 2:**
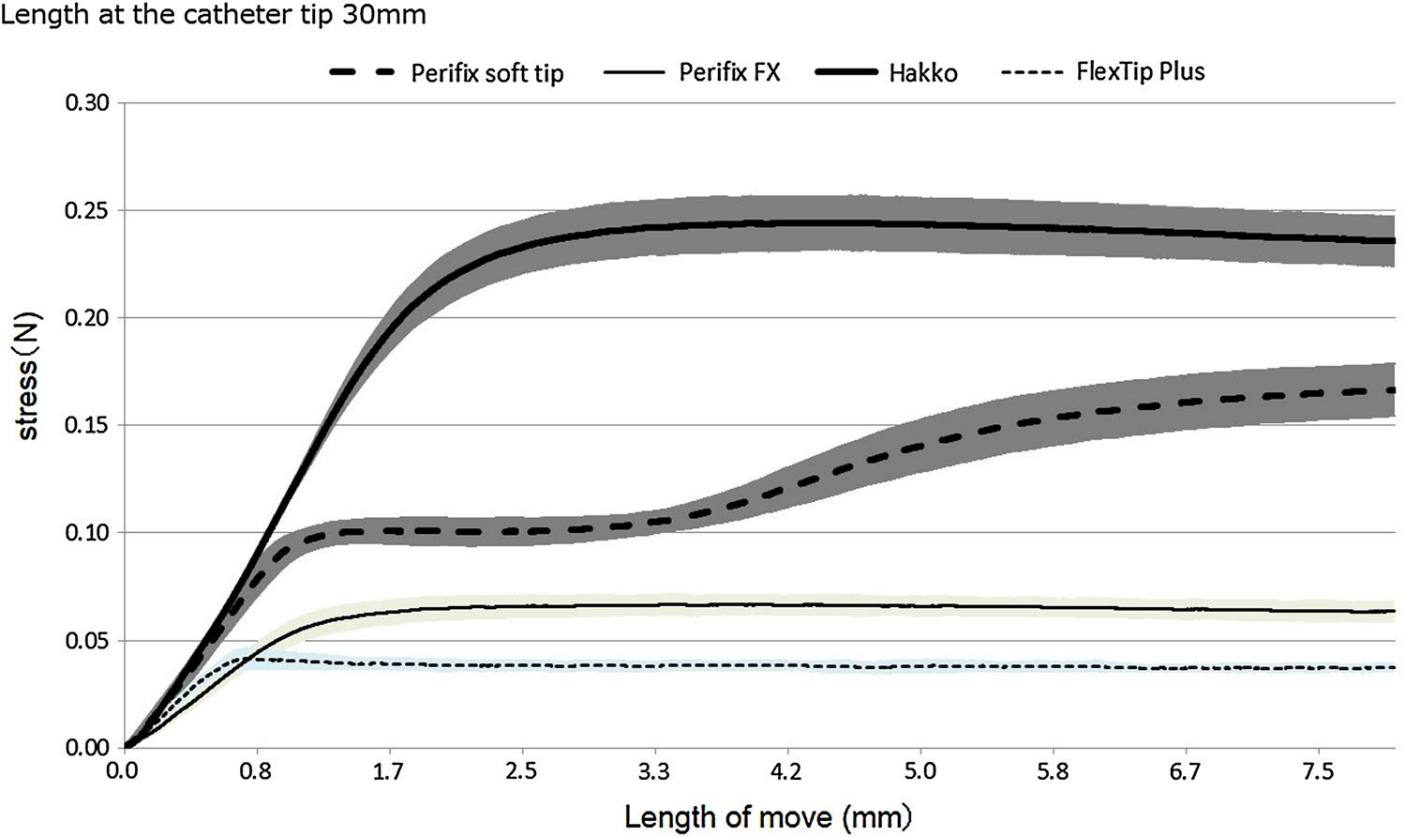
Correlation of the length of movement of the epidural catheters and the stress on the filter from the tips of 30-mm catheters. One catheter was used for each measurement, and five measurements were taken for each catheter type. The mean values were calculated from the obtained measurements and plotted on a graph with standard deviation values according to the catheter type. Graphical forms did not differ by the length of the tip of the catheters except for the value of stress

The force applied to the tip of each of the four types of epidural catheters was evaluated with a Shimadzu Autograph AGX-500 N-111 universal testing machine, and the mean values were plotted on a graph with standard deviation values (Fig. 2). As the Hakko and Perifix Soft tip catheters were advanced, the force applied to the filter rapidly increased. When the Hakko catheter tip protruded by 30, 40 and 50 mm, the force increased to approximately 0.24, 0.15 and 0.1 N, respectively. Meanwhile, when the Perifix Soft tip catheter tip protruded by 30, 40 and 50 mm, the force increased to approximately 0.1, 0.08 and 0.06 N, respectively. Although the force plateaued during the subsequent advance, it gradually increased again after the tip bent. The force transmitted to the tip increased more gradually for the FlexTip Plus and Perifix FX catheters than for the Hakko and Perifix Soft tip catheters. The force transmitted to the tip increased up to approximately 0.06, 0.04 and 0.02 N when the tip protruded by 30, 40 and 50 mm, respectively, with both the FlexTip Plus and the Perifix FX catheters. During the subsequent advance, the force plateaued and did not increase further.

## Discussion

Catheters inserted into the epidural space do not always advance with a straight trajectory and can be displaced, bent, inverted, rotated, etc. In some cases, because a catheter deviates from the intervertebral foramen and causes a unilateral block, the intended anesthetic effect is not achieved, and the discomfort experienced by the patient increases [1–7].

The incidence of deviation varies among studies using radiographs taken with contrast media injected via an epidural catheter, ranging from 1−16 % [19–24]. This may be attributable to the lack of a consistent definition of catheter deviation on radiographs and the fact that the incidence of deviation is likely to be higher when radiographs are used in the evaluation than when the anesthetic effects alone are considered. In the current study, the presence of contrast medium in the paraspinal space or psoas major muscle was considered to indicate deviation of the epidural catheter into the intervertebral foramen in all patients.

In postoperative thoracic epidurography performed at our institution, a relatively high number of cases were difficult to evaluate because enhancement by the injected contrast medium overlapped with a cardiac shadow. Moreover, deviation frequently occurred in the lumbar region, with no difference between the sexes, and the deviation rates according to the insertion site were 10.3 % in the cervical region and 7.9 % in the thoracic region, whereas deviation has been reported to occur in the lumbar region in 20 % of cases [1]. Thus, the present study included only cases of deviation in the region of the lumbar vertebrae, including the intervertebral disk of Th12/L1 at the thoracolumbar junction.

As the technique or maneuver used by the treating anesthesiologist is one of the factors affecting the success of epidural anesthesia, the level of skill is considered to be an indispensable factor [3, 25–27]. The present study revealed significant differences between the groups with respect to the number of years of experience of the treating anesthesiologist. This result is attributed to the fact that some of the treating anesthesiologists had several years of experience because the study period lasted for ≥4 years. In this study, whenever an anesthesiologist with <5 years of experience performed the procedure, a senior anesthesiologist provided supervision. Whenever strong resistance was felt during the insertion of a catheter, or when a patient complained of any abnormality, the insertion of the catheter was stopped. Unless an epidural catheter is inserted under the guidance of fluoroscopy, it is difficult to adjust the catheter’s direction of travel and the length of the inserted portion after entry into the epidural space. The supervision provided by a senior anesthesiologist is presumed to have prevented the number of years of experience from affecting the incidence of deviation into the intervertebral foramen. Bonica et al. reported that even skilled anesthesiologists with 10 years of experience fail in approximately 3 % of cases. Particularly in the case of epidural anesthesia, it is difficult to predict the travel path of the catheter, and this difficulty may be one of the causes of the high failure rate in epidural anesthesia [27]. A similar trend was also shown by Sa’nchez et al., who performed a follow-up examination of radiographs from 90 cases of lumbar epidural catheterization [19]. In Japan, it is also reported that a similar analgesic effect is achieved after surgery when catheterization is performed by either residents (in the late stage of their postgraduate clinical training) or specialists, and that patients are unlikely to experience any disadvantage related to the experience level of the treating anesthesiologist [28]. Regarding the approach to the epidural space, it is generally considered that the paramedian technique facilitates the advance of the catheter toward the head, allows a longer part of the catheter to be placed within the target site, and causes fewer incidents of deviation into the paraspinal space, compared to the median technique [29–31]. However, no significant difference was observed in our study.

Although the FlexTip Plus and Perifix FX catheters, which have internal coils, had significantly lower rates of deviation into the intervertebral foramen after their insertion into the epidural space, the incidence of deviation was significantly higher with the Perifix Soft tip catheter. When force was applied to each of the epidural catheters, the force exerted at the tips of the Hakko and Perifix Soft tip catheters rapidly increased. On the other hand, the force exerted at the tips of the FlexTip Plus and Perifix FX catheters increased to a certain level but then plateaued and did not increase further. This is assumed to be due to dispersion of the force applied to the FlexTip Plus and Perifix FX catheters as a result of their flexibility. Although we speculated that the Hakko catheter would most frequently deviate on the basis of the shape of the curve shown in Fig. 2, the incidence of deviation was actually higher with the Perifix Soft tip catheter. Because the force exerted at the tip of the Hakko catheter increases rapidly and continuously, the treating anesthesiologist may detect resistance and stop advancing the catheter before a certain distance is reached. While the Hakko catheters used in this study are radiopaque, radiopaque catheters are reported to have a high bending stiffness [32]. With a conventional nylon Hakko catheter, which has a lower bending stiffness, the incidence of deviation from the epidural space may decrease. Meanwhile, the force applied to the Perifix Soft tip catheter plateaued at 0.1 N. Because of the flexibility of the tip, the force that is transmitted to it does not increase after the catheter is advanced approximately 1.4 mm. Thus, one reason for the high incidence of deviation may be that the treating anesthesiologist continues to push the catheter forward with a constant force without detecting the increased resistance after that point is reached.

Stiff catheters are reported to be easy to insert into the epidural space [33]. Although the Perifix Soft tip catheter was developed with an emphasis on a straight traveling path in the epidural space, the catheter can be advanced 4–5 cm without coiling in the lumbar epidural space in approximately 13–14 % of cases [4, 20]. It has been reported that the catheter does not reach the intended site in the majority of cases [34, 35]. The advance of the catheter is affected by not only anatomical elements (e.g., blood vessels, adipose tissue, and the septum at the midline of the epidural space), but also by the types of catheters and methods used for paracentesis [36–38]. The rate of catheterizations that achieve a straight travel path is not affected by the stiffness of the catheter itself. Instead, the straightness is more likely to be affected when a stiff catheter is inserted against resistance because the force applied by the fingertips is more easily transmitted to the catheter tip [24, 34]. Meanwhile, the intervertebral foramen is a space where the catheter tip is likely to stay. When a stiff catheter is pushed into the foramen against resistance, the catheter penetrates the areolar tissue and deviates [3]. Previously, we developed models of epidural catheter deviation using the intervertebral foramen in cadaveric specimens, and we found that there were clear differences related to the properties of the catheters. With the Hakko and Perifix Soft tip catheters, the application of a constant force allowed us to easily develop deviation models. However, with the FlexTip Plus and Perifix FX catheters, the reproduction of deviation itself was difficult [18]. Thus, it seems that although the tips of the FlexTip Plus and Perifix FX catheters reach the intervertebral foramen, the force is highly likely to plateau before it becomes large enough to penetrate the areolar tissue and cause deviation. Furthermore, the Perifix Soft tip catheter was the narrowest among all the catheters studied, but the external diameter of the other catheters is almost the same. The gauge of the catheter may suggest the influence of penetrating the areolar tissue easily as well as the stiffness of the catheter.

The FlexTip Plus catheter has been reported to be less likely than other catheters to cause migration into a blood vessel and paresthesia [4, 9–16]. In the present study, although the rates of intravascular migration and paresthesia were also assessed, no significant difference was observed between the catheters. We considered 25 cases in which radiographs showed no evidence of the contrast medium as indicative of the influence of the time course after injection because there was no difficulty injecting local anesthesia through the epidural catheter in the perioperative period, and back-flow of blood from the catheter was not detected. However, we cannot eliminate the possibility of migration into a blood vessel. Assessment by epidurography would have addressed this issue.

The present study has several limitations. First, this was a non-randomized retrospective study conducted at a single institution. Thus, the number of patients was not consistent between the catheter groups, some data relevant to catheterization were not obtained, and perioperative pain management was not consistent. We assume that human factors were unlikely to have affected the results because the same type of catheter was used during each period. However, to obtain more accurate data, multicenter randomized studies may be needed. Second, because the assessment was based on only postoperative plain radiographs, the evaluation of contrast enhancement in the epidural area was limited. Thus, the entire enhanced space could not be visualized, and it was difficult to accurately evaluate the association between the enhanced area and the region or the magnitude of the analgesic effect. Furthermore, although we excluded cases in which the enhanced epidural space was not included in the imaging field of view, cases in which contrast enhancement was not detected because too much time had passed after injection, and cases that could not be evaluated for unknown reasons, it is possible that abnormal findings, such as enhanced subdural space [39–47] and enhanced subarachnoid space [24, 48], might not have been diagnosed sufficiently. Although there have been reports of the usefulness of lateral radiography [2, 3, 49] and computed tomography [6, 36, 50, 51] for the evaluation of contrast enhancement in the epidural space, these imaging studies may be less practical in daily clinical practice because of issues regarding medical costs and procedural simplicity. The present study showed that deviation of an epidural catheter from the epidural space could sufficiently be assessed only with frontal plain abdominal radiographs and that plain abdominal radiography is a simple and useful method to improve the postoperative analgesic effect of epidural anesthesia.

The incidence of epidural catheter deviation into the intervertebral foramen was compared between different catheter types. The incidence of deviation was significantly lower with spiral-type catheters with an internal coil than with other types of catheters. One of the reasons for this appears to be the magnitude of the force that is transmitted to the epidural catheter tip. When epidural catheters are used, we suggest that the incidence of deviation into the intervertebral foramen can be reduced by taking the features of the catheter into consideration and selecting flexible catheters in some cases, instead of inserting stiff catheters against resistance.
